# Low-Dose Mycophenolate Mofetil for Treatment of Neuromyelitis Optica Spectrum Disorders: A Prospective Multicenter Study in South China

**DOI:** 10.3389/fimmu.2018.02066

**Published:** 2018-09-11

**Authors:** Qiao Huang, Jingqi Wang, Yifan Zhou, Hui Yang, Zhanhang Wang, Zhenwen Yan, Youming Long, Jia Yin, Huiyu Feng, Caixia Li, Zhengqi Lu, Xueqiang Hu, Wei Qiu

**Affiliations:** ^1^Department of Neurology, The Third Affiliated Hospital of Sun Yat-sen University, Guangzhou, China; ^2^Department of Neurology, Zhaoqing No. 2 People's Hospital, Zhaoqing, China; ^3^Department of Neuro-Ophthalmology, Zhongshan Ophthalmic Center, Sun Yat-sen University, Guangzhou, China; ^4^Department of Neurology, Guangdong 999 Brain Hospital, Guangzhou, China; ^5^Department of Neurology, Sun Yat-sen Memorial Hospital, Sun Yat-sen University, Guangzhou, China; ^6^Department of Neurology, Second Affiliated Hospital of Guangzhou Medical University, Guangzhou, China; ^7^Department of Neurology, Nanfang Hospital, Guangzhou, China; ^8^Department of Neurology, First Affiliated Hospital of Sun Yat-sen University, Guangzhou, China; ^9^School of Mathematics, Sun Yat-sen University, Guangzhou, China

**Keywords:** neuromyelitis optica spectrum disorders, mycophenolate mofetil, therapy, a prospective study, South China

## Abstract

**Objective:** To evaluate the efficacy and safety of low-dose mycophenolate mofetil (MMF, 1,000 mg/day) treatment of neuromyelitis optica spectrum disorders (NMOSDs).

**Methods:** This study was a multicenter, open, prospective, follow-up clinical trial. The data include retrospective clinical data from the pretreatment phase and prospective data from the post-treatment phase. From September 2014 to February 2017, NMOSD patients seropositive for aquaporin 4-IgG (AQP4-IgG) were treated with low-dose MMF.

**Results:** Ninety NMOSD patients were treated with MMF for a median duration of 18 months (range 6–40 months). The median annual recurrence rate (ARR) decreased from 1.02 before treatment to 0 (*P* < 0.0001) after treatment, and the Expanded Disability Status Scale (EDSS) score decreased from 4 to 3 (*P* < 0.0001). The EDSS score was significantly lower (*P* = 0.038) after the first 90 days of treatment. The serum AQP4-IgG titer decreased in 50 cases (63%). The median Simple McGill pain score (SF-MPQ) was reduced in 65 patients (88%) with myelitis from 17 (range 0–35) to 11 (range 0–34) after treatment (*P* < 0.0001). The median Hauser walking index (Hauser Walk Rating Scale) was reduced from 2 (range 1–9) before treatment to 1 (range 0–7) after treatment (*P* < 0.0001). Adverse events were documented in 43% of the patients, and eight patients discontinued MMF due to intolerable adverse events. Fourteen (16%) of the total patients discontinued MMF after our last follow-up for various reasons and switched to azathioprine or rituximab.

**Conclusion:** Low-dose MMF reduced clinical relapse and disability in NMOSD patients in South China. However, some patients still suffered from adverse events at this dosage.

Clinical Trial Registration: www.ClinicalTrials.gov, identifier : NCT02809079.

## Introduction

Neuromyelitis optica spectrum disorders (NMOSDs) are different from multiple sclerosis and represent a type of B cell-mediated astrocytopathic glial disease (1, 2). NMOSDs mainly affect the optic nerve, spinal cord, and area postrema of the medulla oblongata. NMOSDs have overall high recurrence disability rates. The recurrence rate of NMOSDs is increased in patients with specific biomarkers, such as aquaporin 4-IgG (AQP4-IgG), and the degree of disability increases with the cumulative effects of relapse (3). Therefore, clinicians urgently need to find effective and safe immunomodulatory drugs to treat this condition.

To date, no treatment for NMOSDs has been granted regulatory approval. Because the disease is rare, most relevant clinical studies include small samples and have a retrospective design, and no controlled clinical studies have been reported. Azathioprine (AZA), mycophenolate mofetil (MMF) and rituximab (RTX) are the most widely used agents to treat NMOSDs (4). Our recent study showed that MMF had the same efficacy but fewer adverse events than AZA (5). RTX is more effective than MMF or AZA in preventing relapses and stabilizing disability (6–11). However, the need for regular redosing and monitoring, the cost, and the availability of RTX limit its broad usage in a sizable proportion of NMOSD patients.

MMF has been used in organ transplantation recipients (12) and rheumatoid disease patients (13, 14). Recently, MMF has been gradually introduced as a treatment for neuroimmunological diseases with some success (15–17). Multiple studies have shown that MMF is effective as a treatment for NMOSDs (18–20), and its effect may be better than that of AZA and other traditional immunosuppressive agents (6, 21). The efficiency of MMF is not affected by previous use of other immunosuppressant (6–8, 21, 22). A few studies have reported that MMF is associated with significant adverse events, such as diarrhea, liver enzyme abnormalities, infection, bone marrow suppression, and the occurrence of progressive multifocal cerebral white encephalopathy (6–8, 18, 19, 21, 23).

The dose of MMF used across different institutes for clinical treatment varies, with the dose used in organ transplantations ranging from 250 mg/day to 3,000 mg/day (24, 25). Similarly, in the past, NMOSD patients have received MMF doses of 750–3,000 mg/day, but the safety of the medication has not been fully defined. Therefore, we conducted a multicenter clinical trial to evaluate the efficacy and safety of low-dose MMF for the treatment of NMOSD patients seropositive for AQP4-IgG in South China.

## Materials and methods

### Study design

This study is a multicenter, open, prospective, follow-up, and self-controlled study (ClinicalTrials.gov ID: NCT02809079). The data include retrospective clinical data from the pretreatment phase and prospective data from the post treatment phase. This study was approved by the medical ethics committee of the Third Affiliated Hospital of Sun Yat-sen University (Approval No. the Third Affiliated Hospital of Sun Yat-sen University (2014)2-15 and the Third Affiliated Hospital of Sun Yat-sen University (2015)2-147 No. 1). Eleven patients were enrolled between September 2014 and February 2015 according to the 2006 NMO diagnostic criteria after ethical approval was granted in 2014. Then, the approval was updated following publication of new diagnostic criteria in 2015. The initial 11 patients were reappraised, and all patients conformed to the new criteria. All patients voluntarily provided informed consent.

### Subjects (Figure 1)

Inclusion criteria: (1) conformed to the 2006 NMO diagnostic criteria (26) or 2015 NMOSD diagnostic criteria (4); (2) seropositive for AQP4-IgG; (3) aged 18 years old or older; (4) more than 2 relapses within the 2 years prior to MMF treatment or more than 1 attack in the 1 year prior to treatment; and (5) all other immunosuppressive agents were suspended except for glucocorticoids for more than 3 months.

**Figure 1:**
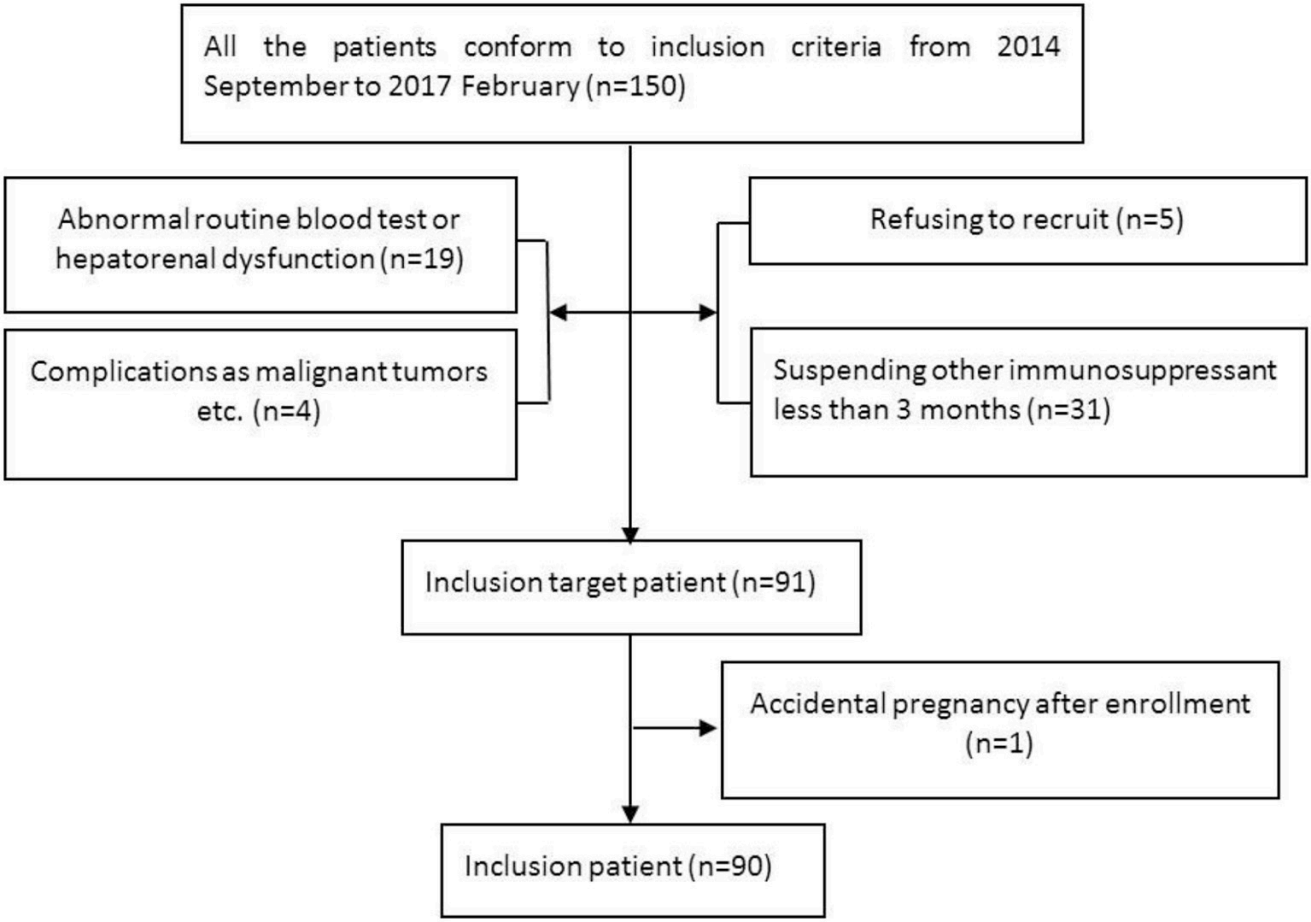
Flow chart of the inclusion and exclusion processes.

Exclusion criteria: (1) transaminase levels beyond the upper limit of normal values; (2) routine blood tests: white blood cell count (WBC) < 4 × 109/L, hemoglobin (HGB) < 110 g/L, and platelet count (PLT) < 100 × 10^9^/L; (3) complications, such as serious circulatory system or other diseases or malignant tumors, immune deficiency, or infection; (4) pregnancy or lactation in women or a recent planned pregnancy for males or females; and (5) a malacetil or glucocorticoid allergy.

AQP4-IgG was tested in the Department of Neurology, the Third Affiliated Hospital of Sun Yat-sen University, using a cell-based assay with HEK293T cells transfected with human AQP4-M23 genes.

### Therapeutic regimen

All patients were treated with MMF plus oral prednisone or methylprednisolone. The total treatment duration for the glucocorticoid was 18 months. The MMF dosage was 500 mg/day for the first 2 weeks and was adjusted to 1,000 mg/day after 2 weeks. Patients who had a relapse were administered intravenous methylprednisolone (500–1,000 mg/day) for 3–5 days, followed by oral prednisone at 30 mg/day (or methylprednisolone at 24 mg/day) for 8 weeks. The prednisone or methylprednisolone dosage was decreased by 5 mg or 4 mg, respectively, every 3 weeks until it reached 10 or 8 mg, respectively, and then was administered every other day.

We prospectively collected clinical features (e.g., disease onset time, relapse time, and recurrences) and evaluated disability, magnetic resonance imaging (MRI) results, immunosuppressant use, the course of drug use, and relevant adverse events.

### Efficacy assessments

The average annual recurrence rate (ARR) before and after MMF treatment was the primary clinically effective outcome. Clinical recurrence was defined as a new symptom of a functional nervous system defect that lasted more than 24 h, an increase in the Expanded Disability Status Scale (EDSS) score of by more than 0.5 points, or MRI results confirming the existence of new lesions.

The degree of disability before and after MMF treatment was the secondary clinically effective outcome and was defined using functional evaluations [i.e., EDSS, SF-MPQ, and the Hauser walking index (Hauser Walk Rating Scale) scores] and structural evaluations (i.e., the longitudinal focus of a spinal T2 sequence on MRI).

### Safety assessments

In this trial, the rate of MMF-relevant adverse events was used as a safety evaluation index. MMF-relevant adverse events were defined as the occurrence of adverse events in line with the characteristics of MMF metabolism. Symptoms must show a clear sequence and improve after suspending MMF. Drug-relevant adverse events, their occurrence times, and the implemented treatment plans were recorded.

### Statistical analysis

Data were analyzed using the R-Studio open source software R-Studio 3.1. The figures were constructed using GraphPad Prism, version 5 (GraphPad Software, La Jolla, CA). A paired Wilcoxon rank sum test was used to compare changes in the titers before and after MMF treatment. A Kaplan-Meier curve was used to compare the incidence of adverse events before and after MMF (95% confidence interval), and the rates were compared using the log-rank test. A meta-analysis was conducted with the R package “metafor” to combine 7 MMF treatment studies of NMOSD patients. The heterogeneity test was performed to detect dispersion across effect sizes, and then the fixed (or random) effects model under no heterogeneity (or under heterogeneity) was constructed to obtain the combined effect. *P* < 0.05 was considered significant.

## Results

### Clinical features (Table 1)

From September 2014 to February 2017, 91 patients with serum AQP4-IgG-positive NMOSDs were enrolled for MMF treatment. Among these cases, 1 discontinued MMF on the fourteenth day due to an accidental pregnancy and experienced a relapse during the second month after delivery. The remaining 90 patients were included in the statistical analysis. Seventy patients were treated only with glucocorticoid prior to MMF treatment. The median therapeutic course was 17 months (range 1–32 months). The other 20 patients received AZA combined with glucocorticoid therapy for a median of 14 months (range 6–66 months) before receiving MMF treatment. These patients had experienced a relapse or adverse events and had stopped AZA more than 3 months prior to beginning MMF treatment. At the last follow-up, 14 cases (15.6%) had switched from MMF to AZA or RTX.

**Table 1:** Clinical characteristics of the 90 NMOSD patients.

**Clinical characteristic**	**Value**
Total patients	90
Female to male ratio	14:1
Age of onset (y)	36 (10–65)
Disease duration before MMF (mo)	52 (1–271)
ARR pre-MMF	1.02 (0.0–19.21)
ARR post-MMF	0 (0–2.44)
EDSS pre-MMF	4.0 (0.0–8.5)
EDSS post-MMF	3.0 (0.0–8.0)
Other autoantibodies, *n* (%)	34 (37.8)
Other autoimmune diseases, *n* (%)	4 (4.3)
Adverse event, *n* (%)	39 (43)
Patients who received AZA before MMF	20
ARR pre-MMF	0.92 (0.09–1.90)
ARR post-MMF	0 (0–2.00)
EDSS pre-MMF	4.0 (3.0–7.5)
EDSS post-MMF	3.0 (1.0–5.0)
Patients who were immunosuppressant naive	70
ARR pre-MMF	1.02 (0–19.21)
ARR post-MMF	0 (0–2.44)
EDSS pre-MMF	4.0 (0.0–8.5)
EDSS post-MMF	3.0 (0.0–8.0)

*ARR, annual recurrence rate; EDSS, Expanded Disability Status Scale score. Data are presented as the frequency (%) or median with range*.

### Efficacy assessments

#### Relapsing

**Figure 2:**
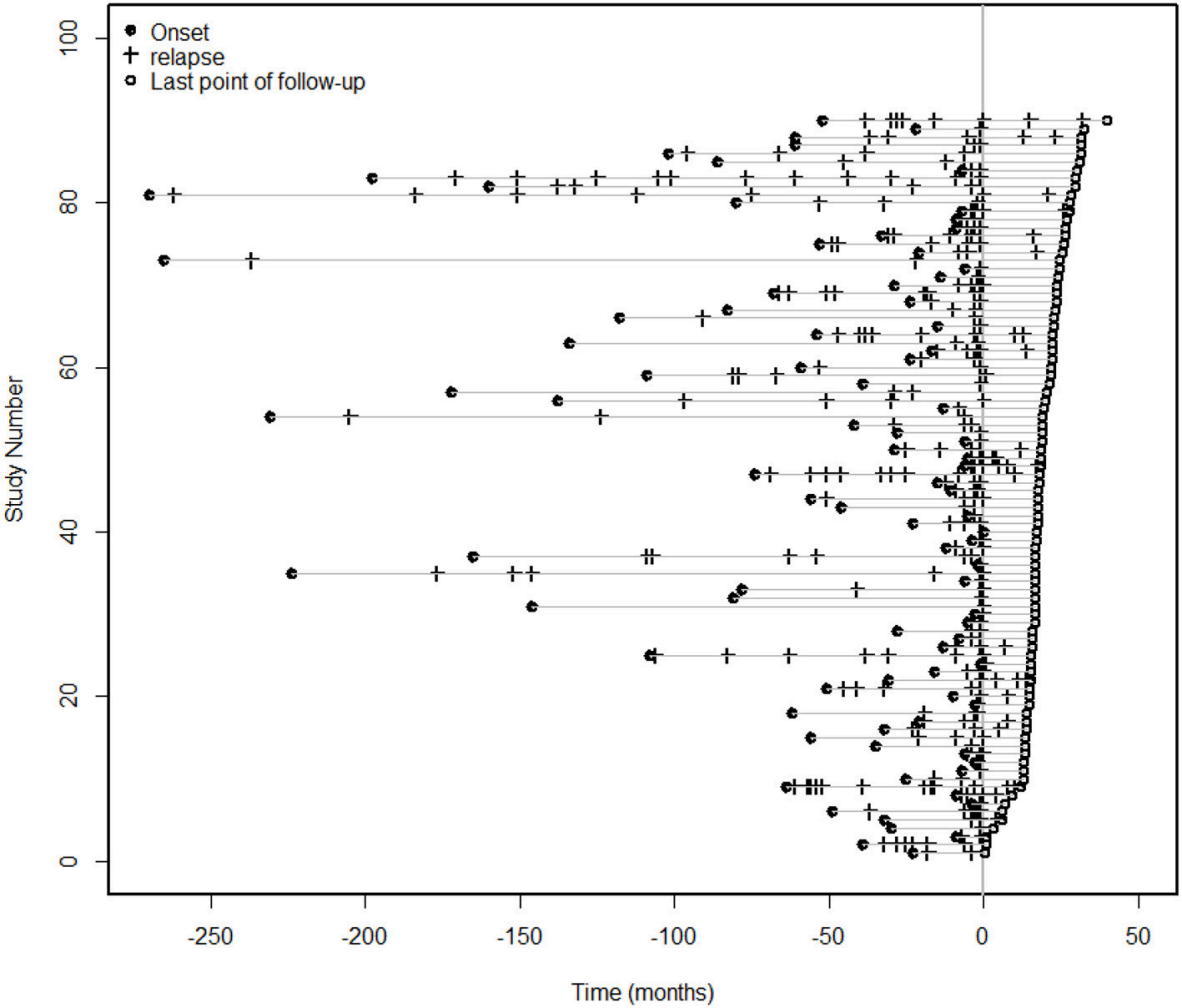
Clinical episodes before and after MMF treatment. For the ARR analysis, we excluded patients with an MMF treatment duration of less than 6 months. The median duration of treatment for the 86 patients was 18 months (range 6–40 months), and the median ARR decreased from 1.02 before treatment to 0 after treatment (*P* < 0.0001). A total of 90% of the patients had a reduction in the ARR, and 73% of the patients had no clinical recurrence.

Ninety patients with NMOSDs were treated with MMF at a dose of 1,000 mg/day. For the ARR analysis, we excluded patients with an MMF treatment duration of less than 6 months. The median duration of treatment for the remaining 86 patients was 18 months (range 6–40 months). The median ARR decreased from 1.02 before treatment to 0 after treatment (*P* < 0.0001); a total of 90% of the patients had a reduction in their ARRs, and 73% patients experienced no clinical recurrence (Figure 2). The mean duration of follow-up after introduction of MMF was 13.5 months, although three cases were followed up for < 1 year. Furthermore, some other studies did not exclude patients with a disease duration of < 12 months (19).

In this study, a subgroup analysis was performed based on whether or not the patient was previously treated with AZA. For the 67 patients who were initially treated with MMF plus glucocorticoid, the median ARR decreased from 1.02 to 0 (*P* < 0.0001), and the ARR decreased in 90% of the patients. Nineteen patients were previously treated with AZA combined with corticosteroids before switching to MMF with corticosteroids. The median ARR in these patients decreased from 1 to 0 (*P* < 0.0001), and the ARR decreased in 91% of these patients. The Cox model was used to correct for sex and age after the Kaplan-Meier survival analysis (Supplementary Figure 2) and showed that the two groups had a significantly lower risk of relapse after treatment with MMF combined with a glucocorticoid (HR = 0.308, 95% CI: 0.209–0.455; *P* < 0.001). However, no significant difference was observed between the two groups (*P* = 0.762).

#### Disability

Of the 90 patients treated with MMF combined with a glucocorticoid, the EDSS score decreased from 4 before treatment to 3 after treatment (*P* < 0.001), and the EDSS score decreased or stabilized in 90% of the enrolled patients. The EDSS score began to decrease after 90 days of MMF treatment, and a significant difference was observed between the groups (*P* = 0.0038). The median Simple McGill pain score (SF-MPQ) was reduced in 65 patients (88%) with myelitis from 17 (range 0–35) to 11 (range 0–34) after treatment (*P* < 0.0001). The median Hauser walking index (Hauser Walk Rating Scale) was reduced from 2 (range 1–9) before treatment to 1 (range 0–7) after treatment (*P* < 0.0001).

#### Serum AQP4-IgG titers

All patients were serum AQP4-IgG-positive. The serum AQP4-IgG titers were measured in 79 patients before and after MMF treatment. The median AQP4-IgG titer was 100 (range 10–320) at the beginning of MMF treatment and dropped to 32 (0–100) at the end of the follow-up period (*p* < 0.001). The titer decreased in 63% of the patients, and 14% (11/79) of the patients had negative antibody results after treatment. Eight patients who were negative for AQP4-IgG antibodies experienced no clinical recurrence after a median follow-up of 16 months (range 13–26 months) (Supplementary Figure 1).

#### Spinal cord MRI

The spinal cord MRI results were compared among 44 patients before and after MMF treatment. The median length of the observed lesion segments was 6 (range 0–17) at the beginning of MMF treatment and dropped to 2.5 (range 0–15) at the end of treatment. In total, 75% of the patients showed a decrease in spinal cord lesions; the lesions were completely absorbed in 32% (14/44) of these patients (Supplementary Figure 1).

### Safety assessments (Table 2)

**Figure 3:**
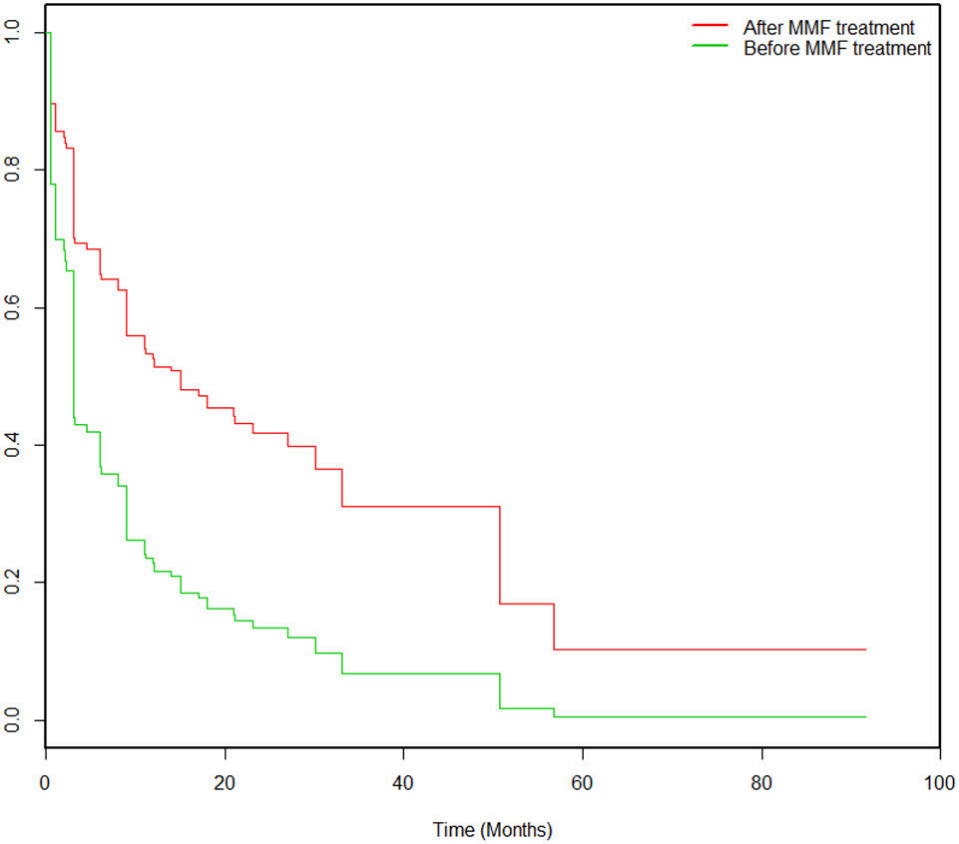
Kaplan-Meier survival analysis pertaining to the probabilities of adverse events pre-MMF and post-MMF in 90 patients. All patients were prescribed MMF at time 0. The incidence of adverse events decreased significantly after MMF administration compared to that before MMF treatment (HR = 0.434, 95% CI: 0.202–0.933; *P* = 0.003).

During the study, 43% (39/90) of the patients experienced MMF-related adverse events, which were concentrated during the period from 14 to 360 days after initiation of MMF treatment. These events included digestive system symptoms (24%, 22/90), infections (23%, 21/90), blood system abnormalities (11%, 10/90), and other adverse effects (hair loss 2%, 2/90; rectal cancer, 1%, 1/90; and renal insufficiency, 1%, 1/90). After correction in the Cox model, the Kaplan-Meier survival (Figure 3) analysis showed that the rate of adverse events associated with MMF combined with glucocorticoid treatment decreased significantly (HR = 0.434, 95% CI: 0.202–0.933; *P* = 0.003).

**Table 2:** Adverse events after MMF treatment in 90 NMOSD patients.

	**Patients with adverse events, *n* (%)**	**Patients discontinuing MMF because of AE, *n* (%)**
Total, n (%)	39 (43)	8 (9)
Gastrointestinal AE, n (%)	22 (24)	2 (2)
Diarrhea	2 (2)	1 (1)
Deranged liver enzymes	18 (20)	1 (1)
Hyperbilirubinemia	2 (2)	0 (0)
Infections, *n* (%)	21 (23)	3 (3)
Respiratory infection	11 (12)	2 (2)
Urinary tract infection	5 (6)	0 (0)
Varicella-zoster virus infection	5 (6)	1 (1)
Hematological AE, *n* (%)	10 (11)	0 (0)
Anemia	6 (7)	0 (0)
Leucopenia	4 (4)	0 (0)
Others, *n* (%)	4 (4)	3 (3)
Rectal cancer	1(1)	1 (1)
Renal insufficiency	1(1)	1 (1)
Hair loss	2 (2)	1 (1)

*AE, adverse event. Data are presented as the frequency (%)*.

In total, 9% (8/90) of the patients terminated MMF treatment, and one patient was converted to AZA treatment. In particular, 2 of the 3 cases of severe pneumonia needed ventilator support. Additionally, 1 patient had “hemorrhagic varicella” in the third month of MMF treatment at a dosage of 1,000 mg/d and died of acute respiratory distress syndrome 4 days later. One patient was treated with MMF for 6 months, had a serum carcinogenic embryonic antigen (CEA) level of 20.90 μg/L, and received an electron colonoscopy indicating a rectal tumor; the pathology of the mass indicated a differentiated adenocarcinoma. MMF was discontinued in this patient, who was subsequently treated with chemotherapy.

### Meta-analysis (Supplementary Figure 2)

A meta-analysis was performed for 7 studies that evaluated MMF treatments in NMOSD patients, including the present study. The median decline in the ARR rate was 0.88, and the rate of stability or decline in the EDSS score was 0.91. In this study, the ARR decreased in 90% of the NMOSD patients, and no clinical recurrence occurred in 73% of the patients. In 90% of the patients, the EDSS score was stable or decreased, and no significant difference was observed between these results and those reported in previous studies.

## Discussion

In this study, the rationale for the use of a lower MMF dose (1,000 mg/day) includes the following. (1) According to our domestic consensus on NMOSD treatment, the recommended MMF dosage is 1.0–1.5 g per day in China (27). (2) Our previous studies have shown that a daily dosage of 20 mg/kg is effective (17). (3) Several studies have reported a higher possibility of adverse events associated with higher MMF doses. A dose-finding study by Squifflet et al. (28) found that a daily dose of 1.0 g was associated with a lower rate of gastrointestinal and hematologic adverse events. Mourad et al. (24) also demonstrated that a 2.0 g dose per day was associated with higher risk of side effects. (4) Finally, economic cost and treatment compliance were taken into consideration for NMOSDs, because the duration of the therapy might be long.

The results of this study demonstrated that a low MMF dose could also effectively reduce the recurrence of NMOSDs and disability. Therefore, we suggest that low-dose MMF can be used in clinical practice, especially in patients in South China with a high relapse risk, high serum AQP4-IgG titers and long segment myelitis, but patients must be closely monitored for adverse reactions. After 90 days of MMF treatment, a significant difference was found in the EDSS scores. Moreover, in this study, the ARRs and EDSS scores were significantly lower in the 20 (22.2%) patients who converted to MMF following AZA treatment, indicating that MMF could be used as a substitute for AZA in patients who did not respond well to AZA. This result is in agreement with the report by Elsone et al. (29), which showed that 46% of NMOSD patients suffered from intolerance or ineffective treatment after AZA treatment. In this study, 27% of the patients still suffered recurrences, which were concentrated within 90–450 days of treatment initiation. Of these patients, 6 cases were changed treatments indicating that some patients experienced poor MMF effects. These unsatisfactory effects may be related to factors such as sex (30, 31), autoantibody production (32), and high serum AQP4-IgG titers (33–35).

Although a lower MMF dose combined with glucocorticoid therapy was used in this study, the adverse events rate (43%) was higher than the rates reported in previous studies. These events, including altered transaminase levels and opportunistic infections, caused 9% of the patients to terminate MMF treatment, which was similar to the proportion observed in another study in which MMF was combined with small-dose glucocorticoids (36, 37). In the meta-analysis (Supplementary Figure 3), the median adverse event rate was 0.29. In this study, the MMF dose was small, but the adverse event rate was higher than the rates reported in previous studies (at an MMF dose of 1,500–3,000 mg/day). We propose that these discrepancies might be related to the following factors: (1) differences in research and study designs, because this study was designed for prospective follow-up and therefore was more likely to record adverse events, and (2) differences in the patient population. For example, a great deal of interindividual variation exists in drug enterohepatic circulation pathways, and the pharmacokinetics of MMF are unstable (38, 39).

We acknowledge that this study has some weaknesses. First, this study did not include a randomized, double blind, cohort design. Additionally, the study lacked a control group. However, the median NMOSD disease duration prior to inclusion was 52 months, and the ARR calculation was based on a long observation period. Therefore, the significant reduction in the relapse rate in our study was most likely evidence of a therapeutic effect and not secondary to recruitment of outlier NMOSD patients with a short history and high relapse rate and subsequent regression to the mean. Another limitation is the short follow-up duration post-MMF therapy, which may underestimate the ARRs of referred patients. Second, no MMF dose stratification study was conducted, and the combination of MMF and glucocorticoid therapy might have affected evaluations of the effects of MMF. Oral steroids may also be beneficial for disease remission. In this study, 70 patients took oral steroids before initiation of MMF treatment. Those patients showed a reduction in the ARR and EDSS score after taking MMF. Third, the myelitis lesion length does not reflect disease severity or recovery and is only taken as a secondary endpoint in the present study. Finally, because we used a standard MMF dosage (1,000 mg per day), we did not monitor the lymphocyte counts. However, in clinical practice, using a target absolute lymphocyte count (e.g., 1,000–1,500 cells/μL) to titrate the dosage is suggested to reduce the adverse event rate of MMS.

In conclusion, low-dose MMF reduced clinical relapse and disability in NMOSD patients in South China. However, some patients still suffered from adverse events at this dosage.

## Author contributions

All authors listed have made a substantial, direct and intellectual contribution to the work, and approved it for publication.

### Conflict of interest statement

The authors declare that the research was conducted in the absence of any commercial or financial relationships that could be construed as a potential conflict of interest.
