# Supplementary material for: Low-Dose Mycophenolate Mofetil for Treatment of Neuromyelitis Optica Spectrum Disorders: A Prospective Multicenter Study in South China
Source: Front Immunol. 2018 Sep 11;9:2066. doi: 10.3389/fimmu.2018.02066 (PMC6143768; doi:10.3389/fimmu.2018.02066)
Supplement: Supplementary file 1 [file Presentation_1.PPTX]

## Slide 1
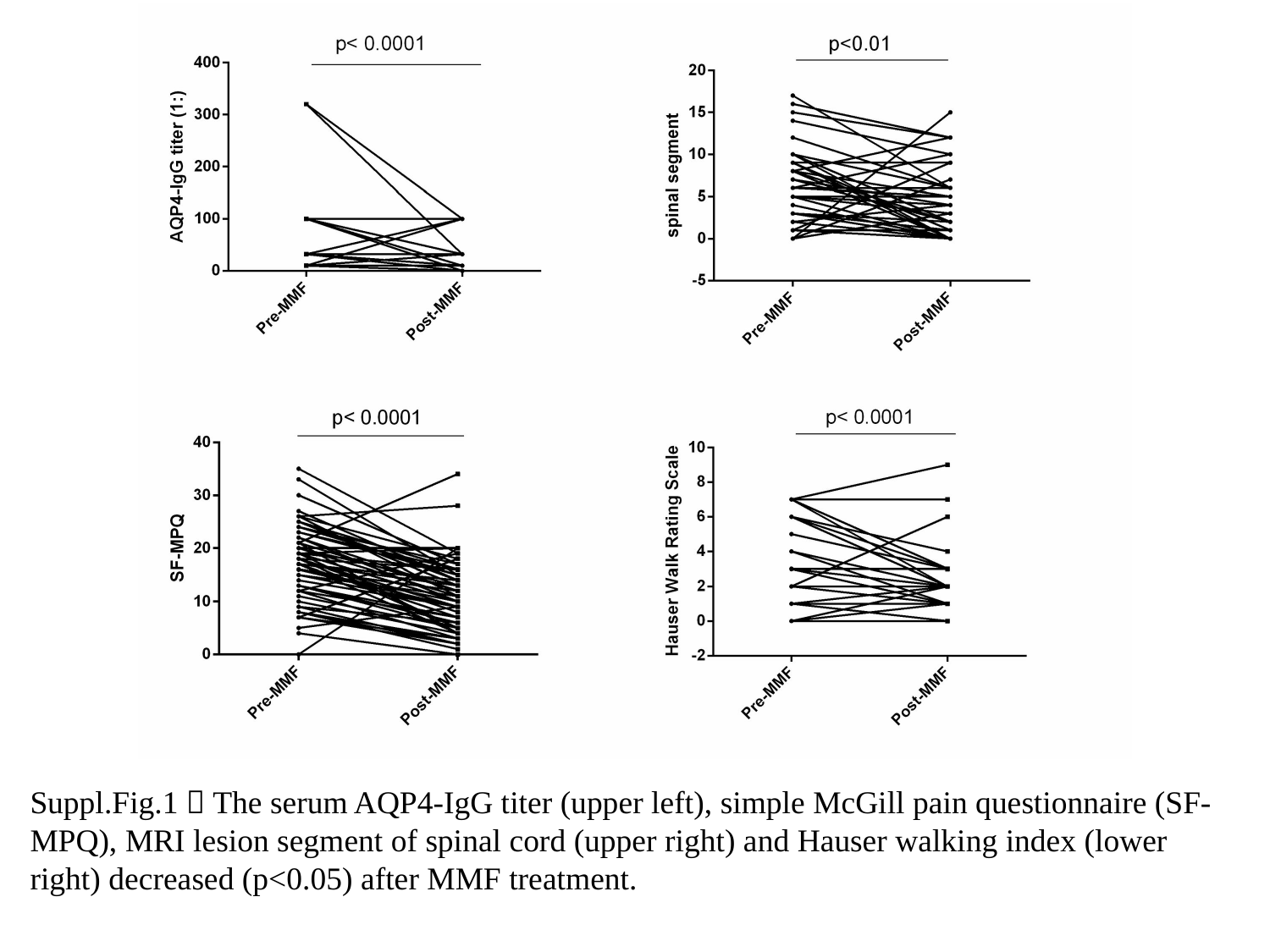

Suppl.Fig.1：The serum AQP4-IgG titer (upper left), simple McGill pain questionnaire (SF-MPQ), MRI lesion segment of spinal cord (upper right) and Hauser walking index (lower right) decreased (p<0.05) after MMF treatment.

## Slide 2
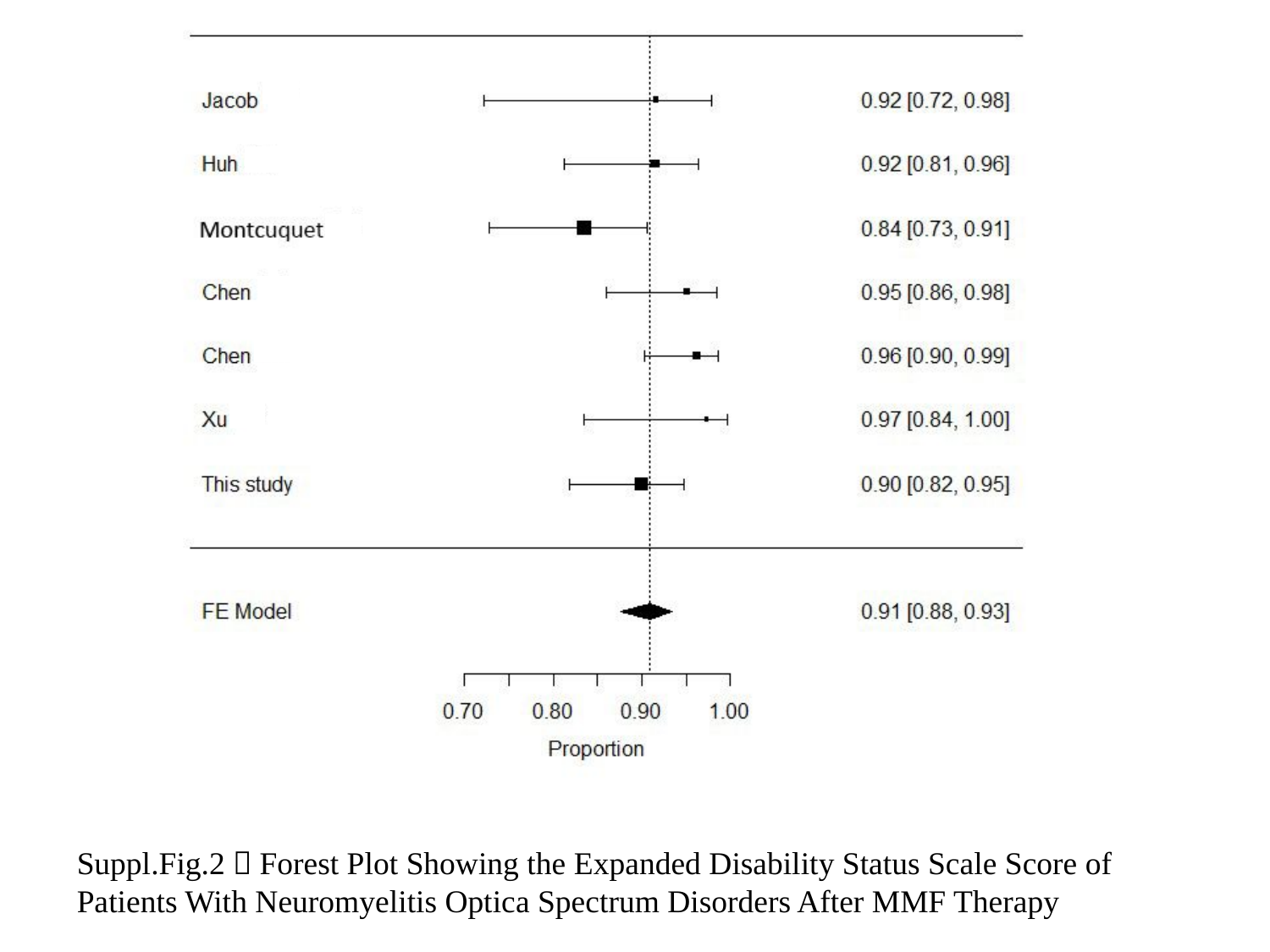

Suppl.Fig.2：Forest Plot Showing the Expanded Disability Status Scale Score of Patients With Neuromyelitis Optica Spectrum Disorders After MMF Therapy

## Slide 3
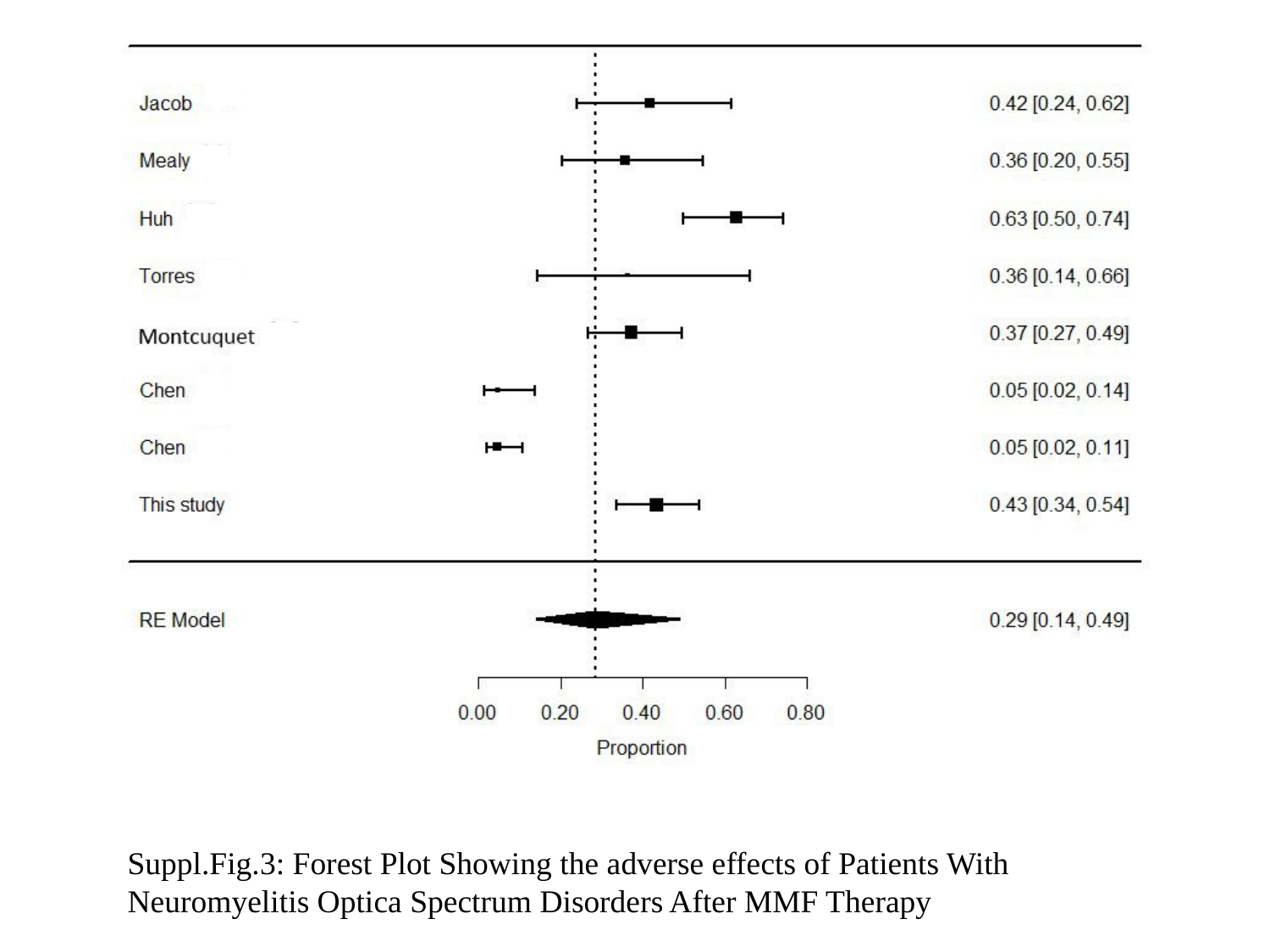

Suppl.Fig.3: Forest Plot Showing the adverse effects of Patients With Neuromyelitis Optica Spectrum Disorders After MMF Therapy
